# Molecular Mechanisms Underlying Antiproliferative and Differentiating Responses of Hepatocarcinoma Cells to Subthermal Electric Stimulation

**DOI:** 10.1371/journal.pone.0084636

**Published:** 2014-01-08

**Authors:** María Luisa Hernández-Bule, María Ángeles Trillo, Alejandro Úbeda

**Affiliations:** Bioelectromagnetics Service, Ramón y Cajal Institute for Medical Research, Ramón y Cajal University Hospital, Madrid, Spain; Columbia University, United States of America

## Abstract

Capacitive Resistive Electric Transfer (CRET) therapy applies currents of 0.4–0.6 MHz to treatment of inflammatory and musculoskeletal injuries. Previous studies have shown that intermittent exposure to CRET currents at subthermal doses exert cytotoxic or antiproliferative effects in human neuroblastoma or hepatocarcinoma cells, respectively. It has been proposed that such effects would be mediated by cell cycle arrest and by changes in the expression of cyclins and cyclin-dependent kinase inhibitors. The present work focuses on the study of the molecular mechanisms involved in CRET-induced cytostasis and investigates the possibility that the cellular response to the treatment extends to other phenomena, including induction of apoptosis and/or of changes in the differentiation stage of hepatocarcinoma cells. The obtained results show that the reported antiproliferative action of intermittent stimulation (5 m On/4 h Off) with 0.57 MHz, sine wave signal at a current density of 50 µA/mm^2^, could be mediated by significant increase of the apoptotic rate as well as significant changes in the expression of proteins p53 and Bcl-2. The results also revealed a significantly decreased expression of alpha-fetoprotein in the treated samples, which, together with an increased concentration of albumin released into the medium by the stimulated cells, can be interpreted as evidence of a transient cytodifferentiating response elicited by the current. The fact that this type of electrical stimulation is capable of promoting both, differentiation and cell cycle arrest in human cancer cells, is of potential interest for a possible extension of the applications of CRET therapy towards the field of oncology.

## Introduction

The exogenous application of electric currents has emerged as an effective therapeutic strategy in the treatment of a number of lesions and ailments [1], [2]. Indeed, electrotherapy has proven effective in relieving pain, promoting blood circulation, reducing the tone of vascular and skeletal muscle and promoting resorption of oedema and joint effusions. Also, in the field of oncology, there is clinical evidence of regression of various cancer types in patients undergoing electrical therapies [3], [4], [5].

The capacitive-resistive electric transfer (CRET) is a non-invasive, electrothermal therapy that applies sine wave electric currents at frequencies between 0.4 MHz and 0.6 MHz, within the radiofrequency (RF) range, and constant amplitudes. These currents induce flow of ions (Na^+^, Cl^−^, K^+^, Ca^+2^, etc.) and dipolar molecules (water, aminoacids, proteins, polysaccharides) in the exposed living tissues. This causes collisions of ions and charged molecules with stationary molecules, which results in tissue heating [6]. The temperature increase is directly proportional to the resistance of the tissue through which current flows [7].

Although capacitive and capacitive-resistive therapies have been classically applied to the treatment of vascular and musculoskeletal injuries [8], new evidence exists indicating that such therapies can also act as adjuvants of chemotherapy or radiotherapy in cancer treatments [9], [10], [11], [12]. In what concerns to CRET specifically, it has been shown to potentiate the action of antitumor agents on the human tongue squamous carcinoma HSC-4 [13]. Also, recent experimental results indicate that CRET effectiveness in cancer treatment may be enhanced by taking advantage of the ability of the radiofrequency current to heat metal nanoparticles embedded in the tumoral tissue [14].

Previous studies by our group have shown that short, repeated stimulation with 0.57-MHz CRET currents at a subthermal dose of 50 µA/mm^2^ can cause a significant decrease, of about 20% below controls, in the proliferation rate of the human hepatocarcinoma cell line HepG2. The effect was proposed to be due to electrically induced arrest in phases S and G1 of the cell cycle in a fraction of the cellular population. These alterations in cell cycle progression were mediated by changes in the expression of cyclins D1, A and B1 and of cyclin-dependent kinase inhibitor p27^kip1^ [15], [16]. Similar effects were observed in human neuroblastoma NB69 cells, in which the same CRET treatment caused cell cycle arrest, accompanied with increased necrosis [17], [18]. On the basis of those results, the present work was aimed to analyze whether the CRET stimulus can also influence cellular and molecular processes involved in HepG2 cell death regulation. Alterations in such processes, along with the observed arrest of the cell cycle, could be responsible for the decline in HepG2 cell population reported by Hernández-Bule et al. [15], [16]. Furthermore, since changes in the levels of cell cycle regulatory proteins have been shown to affect cell differentiation [19] the present study also investigates whether CRET stimulation could exert an influence in the differentiation of HepG2. To that purpose, the levels of expression of alpha-fetoprotein (AFP) and the concentration of albumin excreted to the culture medium were considered as cytodifferentiation markers.

## Materials and Methods

### Cell culture

The hepatocarcinoma cell line HepG2 was purchased from the European Collection of Cell Culture (ECACC, Salisbury, UK). Cells were plated in 75-cm^2^ culture flasks containing DMEM medium supplemented with 10% (v/v) foetal bovine serum, 1% L-glutamine and 1% penicillin-streptomycin, and grown in an incubator (Forma Scientific, Thermo Fisher, Waltham, MA, USA) with a 37°C, 5% CO_2_, humidified atmosphere. Every seven days the cultures were trypsinized and part of the cells were subcultured in flask while the rest of them were seeded at a density of 8.5×10^4^ cell/ml, either directly on the bottom of 60-mm plastic Petri dishes (Nunc, Denmark) or on coverslips placed inside the dishes.

### Electric stimulation protocol

**Figure 1:**
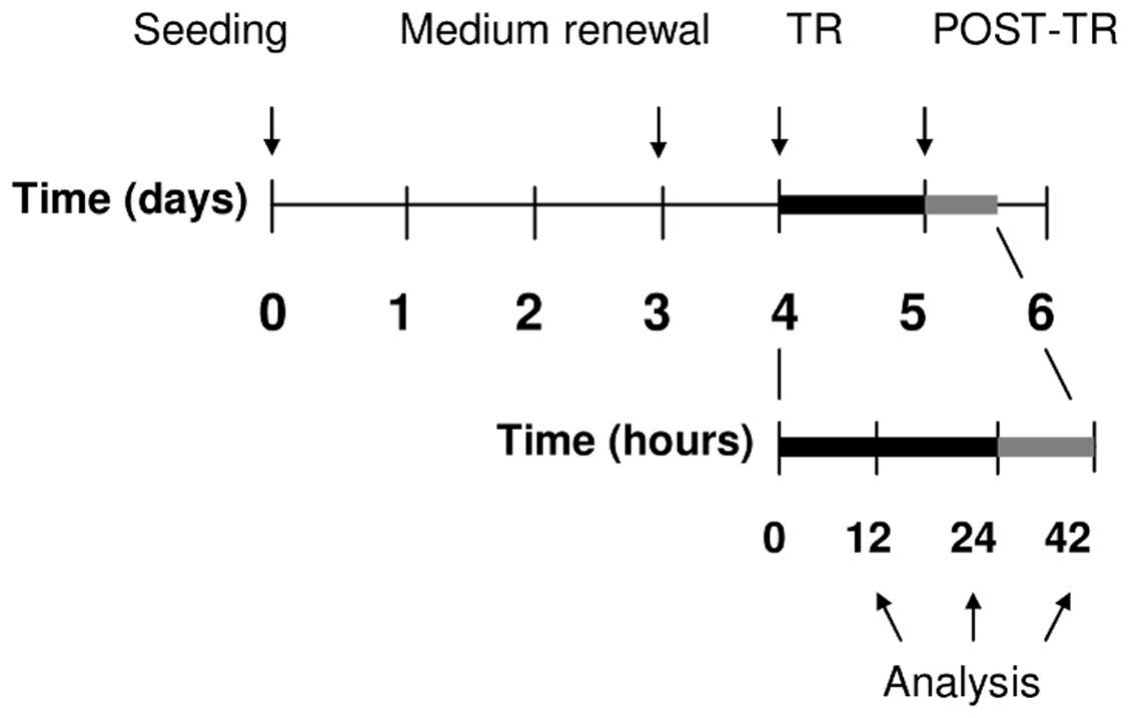
Experimental protocol. Cells were seeded on day 0. On day 4 the samples were either sham-exposed or treated (TR) for 12 h or 24 h with 5-minute pulses of 0.57 MHz, sine wave current at a density of 50 µA/mm^2^. The treatment and sham-exposure intervals were followed by an 18-h lapse of additional incubation (42 h; POST-TR).

The exposure system and the experimental protocol have been described in previous articles [15], [16]. Briefly, exposure to CRET electric currents in Petri dish was carried out through pairs of sterilized, stainless steel electrodes designed ad hoc for in vitro stimulation. At day four after seeding, the electrodes were inserted in the experimental and in the control dishes. The two electrode groups were connected in series to an INDIBA stimulator Mod. M500 (INDIBA®, Barcelona, Spain). On the basis of previously reported data [15], [16], 5-minute pulses of 0.57 MHz, sine wave signal, 50 µA/mm^2^ current density were applied every 4 hours, under controlled conditions of temperature and CO_2_, during 12 or 24 hour intervals (Fig. 1). It has been proven that under these experimental conditions the electrical stimulation does not increase the temperature of the cultures [15]. Simultaneously, the corresponding sham-exposed control samples were kept under the same conditions inside a CO_2_ incubator identical to that housing the exposed samples. In all experiments along the study, the control samples were sham-treated, thus, both terms will be used indistinctly in this text. The ambient electromagnetic field within the incubators was monitored using specific magnetometers for three frequency ranges of interest: static (DC; 0 Hz), power frequency (AC; 50 Hz) and radiofrequency (RF <3 GHz). The mean values obtained were DC: 34.4±3.4 µT rms (Bartington model Mag-03; GMV Associates, San Carlos, CA, USA), AC: between 2 µT and 9.6 µT rms (EFA-3 model BN 2245/90.20; Wandel & Goltermann, Eningen, Germany), and RF: below the detection limit of the magnetometer (PMM 8053 portable field strength meter with external PMM EP-330 E-field probe, both from Narda Safety Test Solutions, Milan, Italy). After electric exposure, groups of 3 to 5 treated and control dishes, depending on the specific experimental procedure, were randomly selected and incubated for an additional period of 18 hours (t = 42 h after treatment onset; Fig. 1). Only those cells grown on the dish surface comprised within the electrode gap (exposed area) were collected for analysis. When appropriate, the cells were grown on two, 12 mm in diameter glass coverslips per Petri dish, placed within the exposed or sham exposed area of the dishes. All experimental procedures were carried out in blind conditions for treatment. The whole methodology and the materials were prior assayed experimentally in order to prevent potential artefacts due to the presence of the electrodes, electrochemical erosion of the electrodes, electrophoretic effects, environmental electromagnetics fields or thermal effects [15].

### TUNEL Assay

Cell death was assessed by terminal deoxyribonucleotidyl transferase (TdT)-mediated dUTP nick-end labelling (TUNEL) assay. Cells were grown in coverslips and fixed with 4% paraformaldehyde at the end of the stimulation interval (t = 24 h after exposure onset) or after 18 additional hours of incubation post-treatment (t = 42 h). The enzyme reaction (1 h at 37°C) was carried out with dUTP-Biotin 10 µM and the enzyme terminal transferase (TdT) 25 U/ml. The preparations were revealed by Avidin and Biotinylated horseradish peroxidase macromolecular Complex (ABC) using diaminobenzidine as substrate, and the cells were counterstained with methyl green. Images were obtained through a digital camera attached to a Nikon Eclipse TE300 microscope using the bright-field. Photomicrographic images were analysed with program Analy-SIS (GMBH, Munich, Germany). A total of 5500 cells at 24-hour experiments, and 14500 cells at 42-hour assays, were analyzed per experimental group in a total of 3 experimental runs per time lapse.

### Flow cytometry

The procedure has been described in detail elsewhere [15]. Briefly, as a qualitative indicator of cell death, the relative fraction of cellular population subG0/G1 (cells with less than stationary cell complement of DNA) was determined through quantification of DNA content by flow cytometry, using Becton-Dickinson FACScan (CellQuest 3.2 Software). Cells were harvested by trypsinization at the end of the treatment or 18 hours after (24 or 42 hours after the stimulation onset) and stained with propidium iodide. In each replicate, 3 cell suspensions per experimental condition, each of them grouping together the cells collected from two Petri dishes, were processed. Three experimental replicates were carried out per time lapse. A total of 20,000 events were acquired per sample and replicate.

### Immunofluorescence

The protocol has been described in detail in a previously published article [16]. Briefly, immunofluorescence assays for alpha-fetoprotein, Bcl-2 and p53 were carried out on cells grown on coverslips. The samples were processed for analysis at different experimental times, depending on the protein of interest. The expression of protein p53 and of alpha-fetoprotein were analyzed during the treatment (t = 12 h), at the end of the treatment (t = 24 hours) and after the post-exposure period (t = 42 hours after exposure onset). Bcl-2 expression was determined at the end of the 24 hour treatment and after 18 hours of post-treatment incubation (42 hours). Anti-alpha-fetoprotein antibody (1∶400, Dako, Glostrup, Denmark), and anti-Bcl-2 (1∶100) and anti-p53 (1∶100, clon PAb 1801) antibodies, both from Biosource (Paisley, UK), were used to detect alpha-fetoprotein, Bcl-2 and p53 antigens, respectively. The samples were studied through fluorescence microscope (Nikon Eclipse TE300). In each experimental repeat of Bcl-2 and p53 immunofluorescence 20 fields were analysed (800 cells per field) per experimental condition. Each analysis was performed in duplicate and repeated at least 3 times for each of the analyzed proteins. Concerning AFP, nearly all cells express AFP immunofluescence, so that quantitative analysis was conducted only through Western Blotting.

### Western Blotting

The protocols for electrophoresis and Western Blotting have been described in detail elsewhere [15]. Briefly, p53 and alpha-fetoprotein expression were analyzed at 12 h, 24 h and 42 h from the beginning of the 24-hour stimulation interval. Anti-alpha-fetoprotein antibody (1∶2000, Dako) and anti-p53 (1∶500, clon PAb 1801, Biosource), were used. Bcl-2 was analyzed at 24 h and 42 h from the stimulation onset using Anti-Bcl-2 antibody (1∶2000; Santa Cruz; Quimigen SL, Madrid, Spain). After stimulation with the 50 µA/mm^2^ current or sham-stimulation, cells were harvested in hypotonic lysis buffer, and proteins were separated and transferred to nitrocellulose membranes. The blots were revealed by ECL-chemiluminescence (GE Healthcare, Little Chalfont, Buckinghamshire, UK) and the bands obtained were evaluated by densitometry (PDI Quantity One 4.5.2 software, BioRad). At least three experimental replicates were conducted for each protein and time interval. β-tubulin was used as loading control.

### Nephelometry

The albumin concentration in the culture media was determined through nephelometry. Media were collected from the dishes at 12 h, 24 h and 42 h from the stimulation or sham-stimulation onset, frozen at −80°C and lyophilized overnight. Lyophilizates were resuspended in sterile bi-distilled water and analyzed with a nephelometer (BN II, Dade Behring, Barcelona, Spain). The albumin antiserum, the internal control, and the buffer for the reaction were purchased from Dade Behring and used following the manufacturer's recommendations. Three experimental replicates were conducted per time interval.

To distinguish the albumin fraction released by the cells from that already present in the culture medium, in each experimental run samples of fresh stock medium were frozen for determination of albumin concentration. The albumin concentration in the fresh medium was measured and subtracted from the values corresponding to the media collected from exposed and sham-exposed samples. The values of released albumin were normalized over the number alive cells in the corresponding samples, quantified by Trypan Blue staining.

### Statistical Analysis

Data were analyzed by two-tailed unpaired Student's t-test using GraphPad Prism Software, San Diego, CA, USA. Differences between samples were considered to be statistically significant at p<0.05, being p the probability of obtaining a result at least as extreme as that actually obtained, assuming that the null hypothesis is true.

## Results

### Assessment of apoptotic response through TUNEL assay

**Figure 2:**
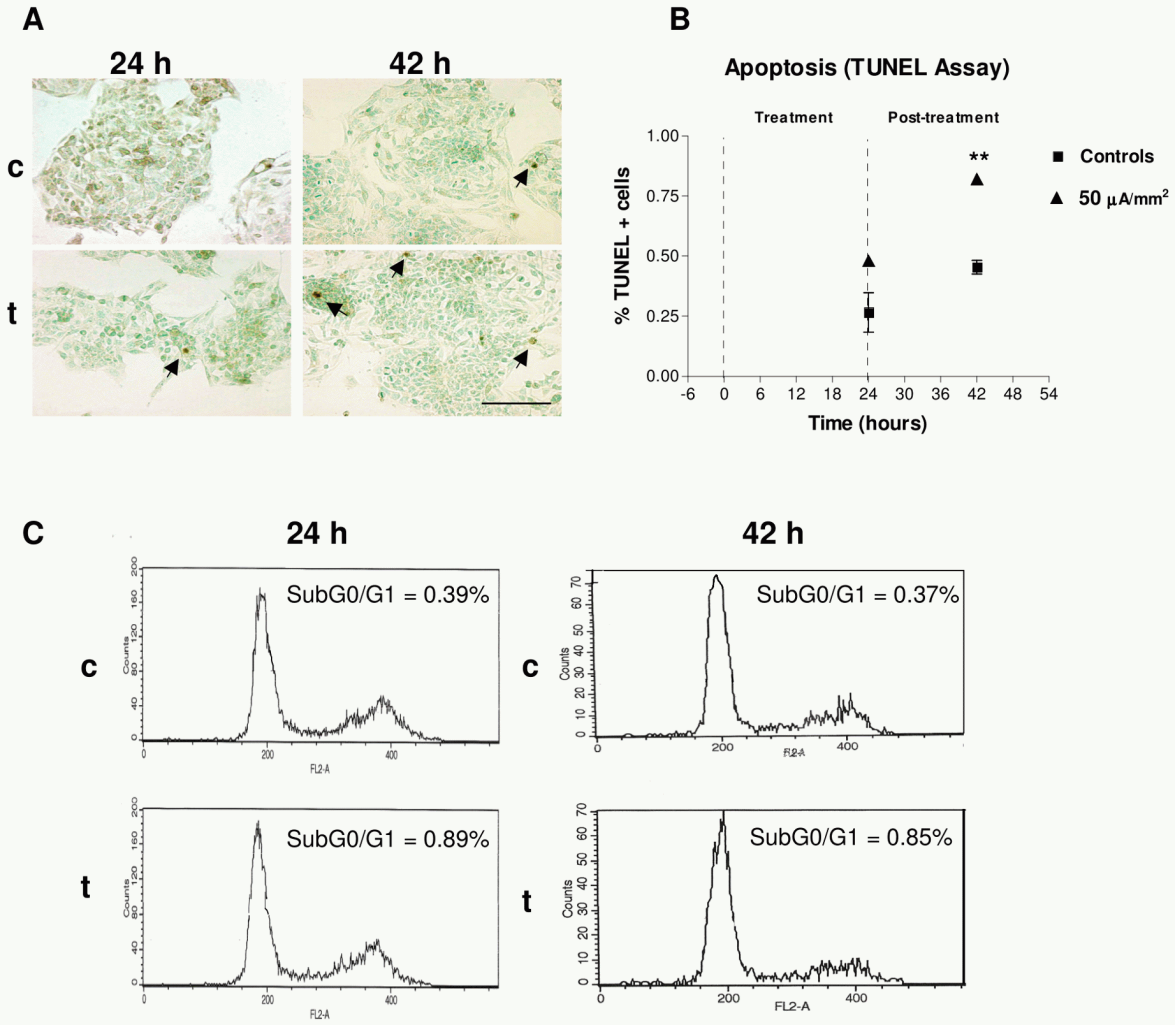
TUNEL assay. (A) Images of apoptosis in sham-exposed controls (c) and in exposed samples, after 24 or 42 h of treatment onset (t). Immunoperoxidase and methyl green staining. Bar  = 100 µm. Arrows point out apoptotic cells. (B) Apoptotic rate at 24 and 42 h: percents of total analyzed cells. Means ± SEM of three experimental replicates; three treated dishes and three controls per replicate. At 42 h the data dispersion in the treated group is very low, so that the error bars are not distinguishable. Asterisks identify statistical significance referring to the corresponding control group; **: 0.001≤P≤0.01; Student's t test. (C) Flow cytometry analysis of the cell cycle in exposed samples and in their corresponding controls. Cells were harvested at 24 h or 42 h after exposure onset and stained with propidium iodide for DNA quantification. Representative results from one single repeat. Each histogram represents the analysis of 20,000 events obtained from the corresponding samples.

At the end of the intervals of treatment/sham-treatment (24 h) and post-treatment incubation (42 h), samples were fixed and analyzed through TUNEL assay. In the sham-exposed cultures the computer-assisted analysis of photomicrographic images showed average rates of apoptosis as low as 0.27% at 24 h, and 0.45% at 42 h. Although the corresponding rates were twice as high in the exposed samples, the differences were statistically significant only for the 42 h cultures, incubated post-treatment (Figs. 2A and 2B).

### Flow cytometric assessment of apoptotic response

The TUNEL technique reveals apoptosis in those cells adhering to the coverslips surface. However, a potentially relevant fraction of apoptotic cells might remain suspended in the culture medium that could not be screened by TUNEL assay. Thus, in order to assess the apoptotic response in the entire cellular population, a flow cytometry assay with propidium iodide was conducted. The analysis of the sub-G1 region confirmed the data in the TUNEL assay (Fig. 2C). Indeed, the cytometric analysis of the control samples revealed apoptotic rates ≤1.5% of the total cell population (adherent cells plus those in suspension). The electric stimulation increased the apoptotic rates with respect to controls, by 12.9%±1.9% (p<0.001) at the end of the 24 h of exposure, and by 73.3%±3.2% (p<0.001) after post-exposure incubation (42 h).

### Effects of the treatment on p53 expression

**Figure 3:**
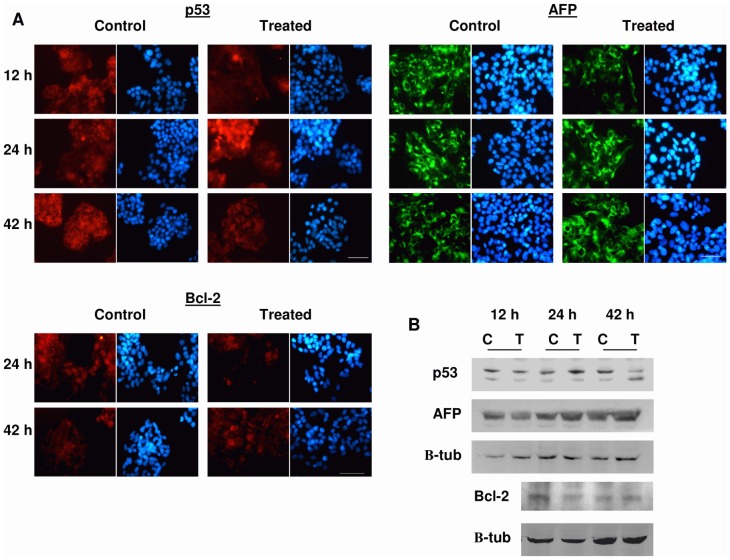
Immunofluorescence and immunoblots for p53, AFP and Bcl-2 expression. (A) Immunofluorescence for p53, and AFP expression during treatment (12 h) and for p53, AFP and Bcl-2 after treatment (24 and 42 h). Samples were processed as described in Materials and Methods. Staining: Alexa Red for p53 and Bcl-2, Alexa Green for AFP, Hoechst 33342 for DNA. Bar  = 50 µm. (B) Representative Blot of p53 and AFP expression during treatment (12 h) and of p53, AFP and Bcl-2 after treatment (24 and 42 h). C  =  control; T  =  treated with 50 µA/mm^2^; p53 and Bcl-2: 100 µg protein/lane; AFP: 30 µg protein/lane. Immunoblot was processed as described in Materials and Methods. β-tubulin was used as loading control.

**Figure 4:**
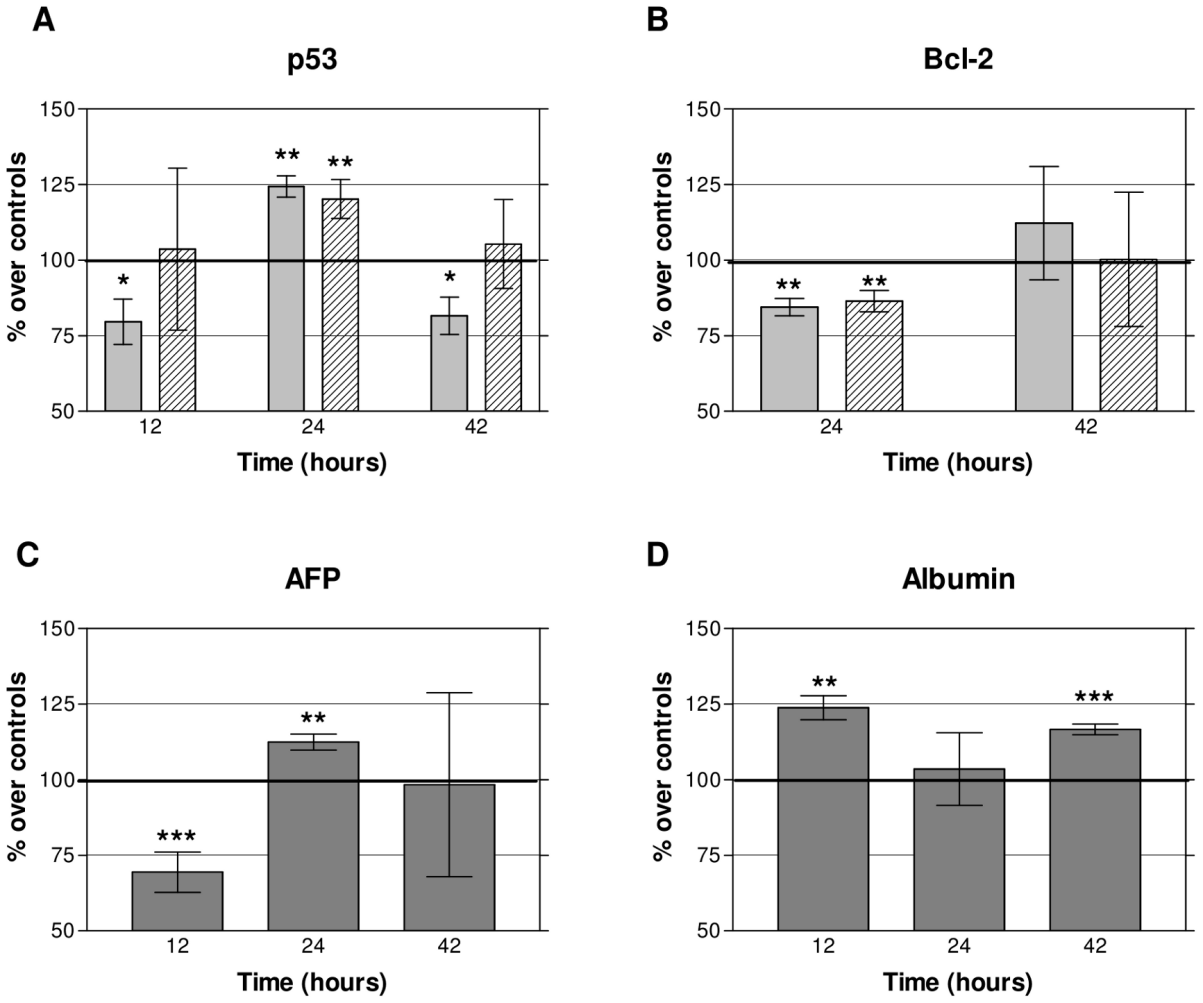
Expression of p53, Bcl-2, AFP and albumin in HepG2 cells. (A) Mean ± SEM values of immunofluorescence and densitometry of Western Blotting for p53 during (12 h) and after treatment (24 and 42 h). Dotted bars: immunofluorescence labeling; hatched bars: blot densitometry. A minimum of three replicates for experimental technique and experimental interval were conducted. Between three and five dishes per experimental group and replicate. (B) Mean ± SEM values of immunofluorescence and densitometry of Western Blotting for Bcl-2 after treatment (24 and 42 h). Same procedures and notations as in A. (C) AFP Western Blotting densitometry during (12 h) and after treatment (24 and 42 h). Means ± SEM of a minimum of three replicates per time interval; five treated samples and five controls per replicate. All Western Blotting values are given as the ratio protein/β-tubulin and expressed as percents over the corresponding controls. D) Albumin nephelometry. Albumin concentration (ng/living cell) released into the culture medium by the cells during (12 h) and after treatment (24 and 42 h). Means ± SEM of three replicates per time interval; two treated samples and two controls per replicate. Asterisks identify statistical significance referring to the corresponding control group; *: 0.01<P<0.05, **: 0.001≤P≤0.01, ***: p<0.001 (Student's t-test)

The immunofluorescence assay revealed that after 12 h, both the treated and control cells showed a dotted or diffuse pattern of cytoplasmic distribution of p53 (Fig. 3A) whereas the proportion of p53+ cells was significantly reduced in the treated samples (Figs. 3A and 4A), though this effect was not detected by Western Blot analysis (Figs. 3B and 4A). At the end of the 24 h treatment the immunofluorescence analysis of the control samples showed that the p53 labelling remained at the cytoplasmic location, whereas in electrically stimulated cells p53 had a preferentially nuclear distribution, indicating that treatment activates p53 (see review by O'Brate and Giannakakou, [20]). The immunofluorecence data showed that the average ± SEM proportions of p53+ cells in controls were 33.61%±3.73%, 40.07%±5.41% and 44.82%±1.50% at 12 h, 24 h and 42 h, respectively. Both immunofluorescence analysis as immunoblot revealed significant increases of p53 levels, of approximately 20% over controls, at the end of the treatment (Figs. 3B and 4A). After 18 hours of post-treatment incubation (t = 42 h) the immunofluorescence analysis showed a significant decrease of p53 expression in the treated samples with respect to controls (Figs. 3A and 4A) which, again, was not detectable by Western Blotting (Figs. 3B and 4A).

### Effects on Bcl-2 expression

After 24 h, both the treated and control cells showed a cytoplasmic distribution of Bcl-2, preferentially located at mitochondrial or reticular levels, although some labelling was also present in mitotic nuclei. However, at that time the proportion of Bcl-2+ cells revealed by immunofluorescence in control samples (mean ± SEM  = 32.55% ± 11.25%) was significantly reduced, by 16%, in the exposed cultures (Figs. 3A and 4B). Such effect was confirmed by Western Blot analysis, which revealed a significant, 13.6% reduction in the Bcl-2 expression at the end of the 24-hour treatment (Figs. 3B and 4B). After 18 h of post-exposure incubation in the absence of the stimulus (t = 42 h) the levels of Bcl-2 expression in the treated samples had recovered, reaching values that did not differ significantly from those in controls (Figs. 3B and 4B).

### Effects on the expression of intracellular alpha-fetoprotein

Hepatoma cells can synthesize various tumor-related proteins, such as AFP [21]. Specifically, AFP, which is synthesized by transformed hepatocytes with high-grade malignancy, is known to be present in HepG2 cells [22]. In fact, all cells in the images taken from our HepG2 cultures expressed AFP to a greater or lesser extent. Our qualitative immunofluorescence analysis of AFP labelling showed that, as observed by other authors [23], AFP tends to concentrate in large vesicles at cytoplasm location (Fig. 3A). Such analysis also showed that compared to the respective controls, the AFP+ labelling was reduced after 12 h of treatment, and increased at 24 and 42 h. These results were reinforced by those from Western Blot quantitative densitometric analysis, which revealed significant decreases in AFP expression, of around 20% below controls, after 12 h of treatment, and increased levels of AFP at the end of the 24 hour exposure (Figs. 3B and 4C). Nevertheless, such reversal of the response elicited during the treatment could be transitory in the absence of electric stimulus, since after 18 h of incubation post-treatment (42 h) the differences between the stimulated samples and their controls did not reach statistical significance (Fig. 4C).

### Effects on the albumin concentration in the culture medium

Serum albumin is a blood plasma protein that participates in the regulation of osmotic pressure in plasma and in transportation of endogenous and exogenous substances. Albumin secretion is recognized as one of the phenotypic features that are specific of mature hepatocytes [24], [25]. Nephelometry of control samples revealed mean ± SEM values of 1.14±0.21, 0.77±0.04 and 0.89±0.07 ng albumin per living cell at incubation times of 12 h, 24 h and 42 h, respectively. The analysis of samples treated with CRET for 12 h revealed a significant increase, of approximately 25% over controls, on the concentration of albumin released to the medium by the cells (Fig. 4D). This effect, which was no longer detectable twelve hours later, after the total 24 h of treatment, reappeared at 42 h, after 18 h of post-treatment incubation.

## Discussion

Previous studies by our group have shown that short, repeated stimuli with 0.57-MHz electric currents at subthermal levels induce a decrease in the proliferation rate of HepG2, due at least in part to blocking of the cell cycle in an electrically sensitive fraction of the cellular population. Such blocking is mediated by changes, occurring during and after treatment, in the expression and activation of inhibitor p27 and of cyclins D1, A, B1, proteins that regulate the cell cycle progression ([15], [16]; see Table 1). Pathways as that of MEK/ERK1/2, which has been shown sensitive to the action of electric and electromagnetic fields [26], [27] are likely to be involved in the response of those regulatory proteins.

**Table 1:** Summary of previously published data and present results.

		Treatment		Post-treatment	
Time after exposure onset		12 h	24 h	42 h	48 h
Proliferation [15]		-	▾**	▾**	▾*
Cell cycle phases [15]	G0/G1	-	▴**	-	-
	S	▴*	▾*	-	▾*
	G2/M	-	-	-	-
Cyclin expression [16]	D1	▴*	▴*	▾*	ND
	A	▴***	▾**	▾**	ND
	B1	-	▾*	-	ND
p27 expression [16]		-	▴*	▾**	ND
Apoptosis		ND	-	▴**	ND
p53		▾*/-	▴***	▾*/-	ND
Bcl-2		ND	▾**	-	ND

**Figure 5:**
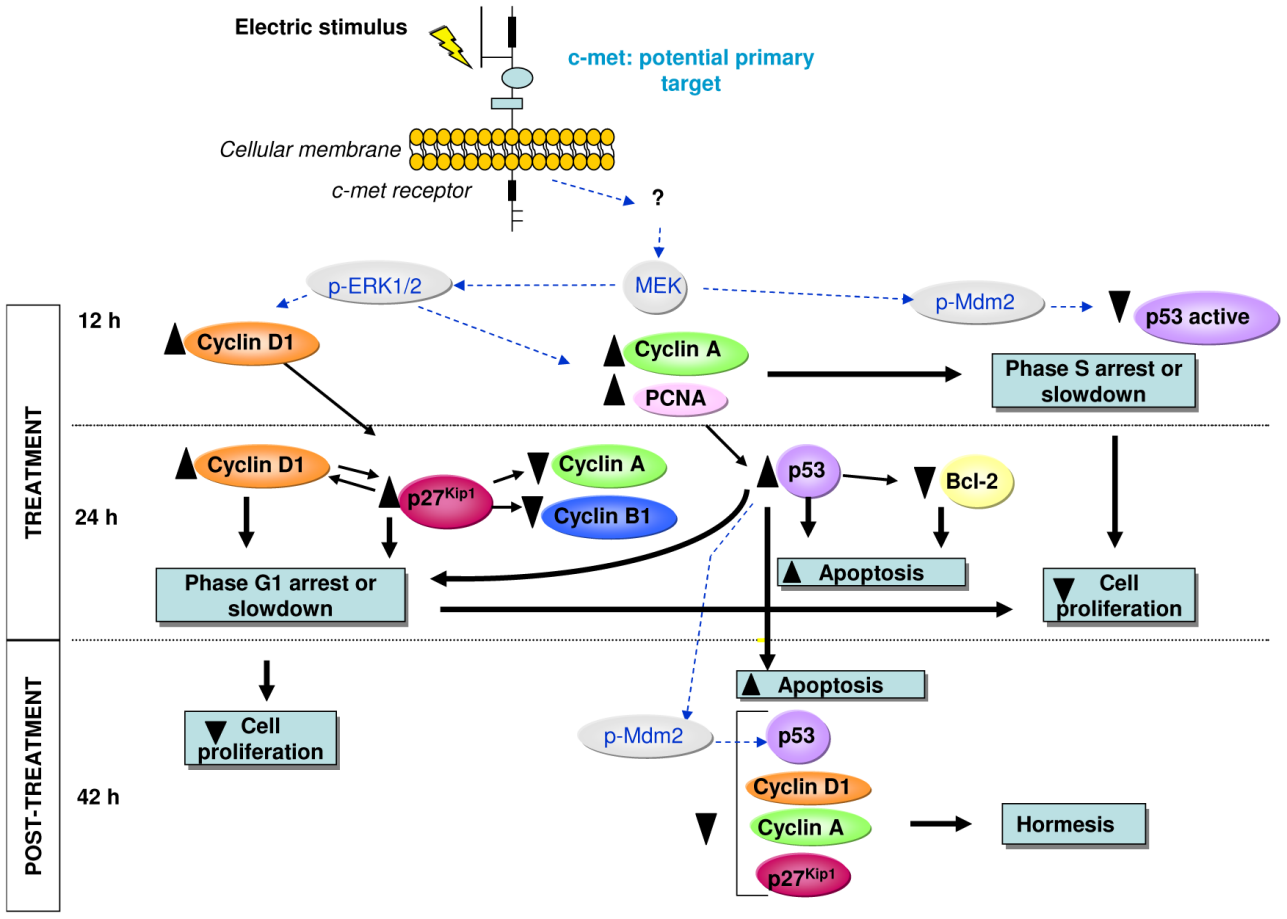
Schematic representation of the cascade of events triggered by CRET stimulation. Sequential progression of the response during (12 h) and after treatment (24 and 42 h). Blue dotted arrows: proposed action pathways. Black arrows: confirmed pathways; ▴: statistically significant increases; ▾ statistically significant decreases (see also Table 1).

▴: statistically significant increase; ▾: statistically significant decrease; ND: no data; -: no significant (0.05<p); *: 0.01<p<0.05; **: 0.001≤p≤0.01; ***: p<0.001 (Student's t-test); ▾*/-: statistically significant decrease of active p53 only, no change in total p53 (see Figs. 4A and 5); [15] and [16]: previously published data, see References.

In order to search for further potential causes of the observed anti-proliferative response, the present study investigated whether significant alterations in cellular and/or molecular processes regulating cell death and survival might contribute to the CRET effect. Although it has been reported that CRET stimulation induces necrosis in human neuroblastoma cells NB69 [17], [18], in HepG2 the observed decrease in the number of alive cells is not accompanied with increased rates of necrosis [15]. Thus, the present work investigated the potential apoptotic action of CRET in HepG2. The results of flow cytometry and TUNEL assay revealed a very low apoptotic rate in controls (<1.5% of the cell population) which was significantly increased in the experimental samples after 18 h of post-treatment incubation (t = 42 h; Fig. 2). This electrically induced proapoptotic effect is consistent with observations by other authors reporting increased apoptosis in rodent and human cells exposed to electric fields [28], [29]. However, given the low rate of spontaneous apoptosis in our samples, it is unlikely that the proapoptotic response to CRET plays a significant role in the decline of cellular population induced by the treatment.

On the basis of the foregoing, we investigated the possibility that CRET induced additional alterations at the molecular level, affecting processes intervening in cell survival and cell cycle regulation. To that end, we examined the effects of the treatment on the expression of two proteins associated with those processes: p53 and Bcl-2. The product of the tumor suppressor gene p53 is a member of the pathway of response to DNA damage and is involved in cell cycle regulation (see Carvajal and Manfredi [30] for a review). Under normal conditions, protein p53 remains in its inactive form and is located predominantly in the cellular cytoplasm. In the event of DNA damage or cell cycle alteration, p53 is transferred to the nucleus, where it becomes functional and capable of inducing apoptosis or cell cycle arrest, as it acts as a transcription factor of genes such as p21 and GADD45 (see review by Liang and Clarke [31])

The results of the study of the expression and location of p53 during and after CRET treatment indicate that this protein could intervene in the apoptotic response detected at 42 h from the starting of exposure, as described in the preceding paragraph. At 12 h of treatment, the immunofluorescence assay showed a statistically significant decrease, of 20% below controls, in the amount of nuclear, active p53. However, Western Blotting did not reveal significant changes in the total levels of p53 in treated samples. Taken together, these data are indicative that, initially, CRET stimulation blocks the activation, but not the expression of protein p53 (Figs. 3 and 4A). This inactivation of p53 could be mediated by Mdm2 phosphorylation, since the electrically induced activation of Mdm2 could allow exporting of p53 from the nucleus to the cytoplasm, which would explain the decrease of nuclear p53 detected by immunofluorescence (Fig. 5). Such translocation would affect p53 activation without changing its overall cellular levels, as shown by Western Blot densitometry at 12 h of treatment (Figs. 3B and 4A). As for the Mdm2 activation, it could be mediated by processes that involve the MEK/ERK pathway and are specific to hepatocytes in general, and to HepG2 in particular [32] (Fig. 5). The hypothesis that the activation of the MEK/ERK is relevant to the cellular response to CRET was already proposed in previous studies by our group, since the activation of this pathway also seems to be involved in the CRET induced arrest of the cell cycle in the line HepG2 [16].

At the end of the 24 h treatment, the expression of p53 in the exposed samples was significantly increased (25% over controls). This, together with the increased presence of p53 in the nucleus of the treated cells (15% over controls), would be indicative of electrically-induced expression and functionality of this protein, which could account for the apoptotic response observed 18 hours after the end of treatment (42 h). Stimulation of p53 at 24 h of treatment might also intervene in the arrest or slowing of phase G1 of the cell cycle, detected at the end of exposure and 18 h after, since p53 can activate cyclin-dependent kinase inhibitors (CKI) that are effectors of cell-cycle arrest. The increased expression and activation of p53 at the end of the 24 h could be caused by prior alterations in the regulation of cell cycle progression, in particular by those related with overexpression of cyclin A and arrest of the S phase at 12 h of treatment, as reported in earlier studies ([16] and Fig. 5). Indeed, a number of abnormal-proliferation signals have been shown to activate p53, which acts as a transcription factor of genes involved in cell cycle arrest [33].

The activation and overexpression of p53 detected at the end of the exposure reverted after 18 hours of post-treatment incubation, showing a statistically significant decrease with respect to controls in activated p53, but not in the total expression of this protein. Such decrease could reflect a negative feedback caused by the protein overexpression registered at the end of the treatment. As indicated above, Mdm2 inhibits p53, but in turn, overexpression of p53 can activate Mdm2 [34], resulting in a subsequent decrease in p53 levels. This negative feedback is consistent with our present results. Indeed, the increased expression of p53 at 24 h of treatment, due to the anomalies observed in cell cycle progression at 12 h, would activate Mdm2, resulting in the inhibition of p53 and subsequent reduction of the protein levels, detected 18 hours after the ending of stimulation. Also, reduced levels of p27 and cyclins A and D1 were detected after the post-treatment interval. This decrease in cell cycle regulatory proteins previously overexpressed in the presence of the treatment might be the result of an adaptive process or hormesis, through which the culture would counterbalance the cytostatic action of the treatment and regain homeostasis (Fig. 5).

In order to deepen the study of potential molecular alterations in cell survival processes, we analyzed the expression of the anti-apoptotic protein Bcl-2. In most cases, overexpression of Bcl-2 contributes to expansion of tumor cells by prolonging cell survival through the delay or arrest of apoptosis, rather than by promoting proliferation (see review by Tomek et al. [35]). While some authors have reported that certain clones of the HepG2 line do not express Bcl-2 [36], [37], our immunofluorescence data reveal that the HepG2 cells used in the present study do express that protein (Fig. 3A). In fact, the low rate of apoptosis in our control samples may be related to the ability of these cells to express Bcl-2, since tumors with high levels of Bcl-2 expression usually show low rates of apoptosis [38].

Our results show that after 24 h of treatment, Bcl-2 expression decreased significantly with respect to controls (Figs. 3 and 4B). This subexpression of Bcl-2 could be induced by the above described activation of p53, since p53 has been shown to block Bcl-2 transcription [39], [40], [41] (Fig. 5). Besides, this decrease in Bcl-2 could induce apoptosis initiation in the electrically stimulated cells, which may explain the cell death increase detected after the 18 h interval of post-exposure incubation. At that time (42 h) the subexpression of Bcl-2 observed at the end of the 24 h of treatment was no longer detectable (Figs. 3 and 4B). This transience in the response of Bcl-2 could plausibly explain the transience of the pro-apoptotic effect elicited by the electric stimulus.

Subexpression of cyclin A, accompanied with overexpression of p53, p27 and cyclin D1 has been observed associated to cytodifferentiation processes [42]. Since our samples exhibit those very changes in the expression of these proteins at the end of treatment with CRET (Fig. 5), we investigated whether the electric stimulus also induces changes in the differentiation of HepG2. To that purpose, we examined the expression of two typical markers of liver cell differentiation: alpha-fetoprotein and albumin. AFP is a liver specific glycoprotein that expresses primarily at the foetal liver and the placenta, and since it overexpresses in 60%–70% of hepatocellular carcinoma cells, is used as a tumor marker in hepatocarcinoma diagnosing [43]. The expression of albumin, a major plasma protein, is currently used as a specific marker of mature hepatocytes [24]. Although albumin expression varies among different hepatocarcinoma types, high expression levels of this protein are generally observed in well-differentiated hepatocellular carcinomas [25].

After 12 h of exposure, a consistent, statistically significant decrease in the levels of intracellular expression of AFP was observed. However, this potential cytodifferentiating effect was no further detected, neither at the end of the 24 h of treatment nor after the post-treatment interval (42 h). Moreover, at the end of the treatment the levels of AFP expression were significantly increased over those in controls, which could reflect a cellular reaction to the strong differentiating stimulus corresponding the first hours of treatment (Fig. 4C).

The quantification of albumin released by the cells to the culture medium after 12 h of treatment revealed a statistically significant increase in its levels, which supports the results on AFP expression after the same exposure interval and confirms the cytodifferentiating action of CRET. The same increase in albumin was also observed after the post-treatment period (42 h). By contrast, at the end of the 24 h of treatment the albumin content in the treated samples did not differ from that in controls (Fig. 4D). This lack of effect could be caused by saturation of the response, due to the strong stimulation of albumin synthesis and/or release elicited during the early hours of treatment.

The biophysical mechanism responsible for transducing the applied electrical stimulus to the cytochemical responses described here is yet to be identified. A possible transduction pathway could involve c-met, the receptor of the hepatic growth factor (HGF). It has been described that extracellular stimuli like HGF are involved in the regulation of cell proliferation in hepatocellular carcinoma [44], as well as in the expression of albumin and alpha-fetoprotein [45]. Several hepatocarcinomas, including the line HepG2, are known to over-express c-met [46], [47]. Therefore, CRET-induced alteration in the HGF receptor c-met or in the receptor-mediated signal transduction pathway, could account for the observed inhibition of cell proliferation and for the albumin and AFP differentiating responses (Fig. 5). Furthermore, ERK1/2, the signalling event down-stream the HGF/met pathway [48] could be altered by CRET, causing the cytostatic/oncostatic response observed in HepG2.

## Conclusions

In sum, the herein reported results show that at 18 hours after the end of a 24-h interval of stimulation with subthermal, 5-minute pulses of radiofrequency CRET signal, a statistically significant increase in the apoptotic rate of HepG2 was observed. However, since this increase in cell death only affected a minor fraction of the cell population, its estimated contribution to the antiproliferative action of the treatment would be of little relevance. Also, significant changes were found at the end of the 24-h treatment in the expression of p53 and Bcl-2. Such changes, which could be responsible for the subsequent increase in the rate of apoptosis as well as for the cell cycle arrest observed in previous studies, would ultimately lead to the also reported decrease in cell proliferation. Additionally, a significant decrease in the expression of the dedifferentiation marker AFP, together with an increase in the albumin concentration in the medium were obtained, which indicates that CRET treatment also could transiently promote cell differentiation in HepG2.

As a whole, our results show that repeated stimulation with CRET inhibits proliferation of HepG2 by arrest of cell cycle progression, and modulates the expression of a number of molecular markers of cell differentiation and malignancy, which suggests that CRET treatment may channel hepatocellular carcinoma cells towards normalization. These results, taken together with those from previously published studies conform a block of information that may be crucial to the development of emerging cancer treatments based on the application of electric or electrothermal therapies.
